# Split Skin Grafting for Leg Ulcers Complicating Rheumatoid Arthritis

**DOI:** 10.4103/0974-2077.41158

**Published:** 2008-01

**Authors:** Reena Rai, M Aruchamy, Chakravarthi R Srinivas

**Affiliations:** *Department of Dermatology and Plastic Surgery, PSG Hospitals, Coimbatore, Tamil Nadu, India*

**Keywords:** Leg ulcer, Rheumatoid arthritis, Cyclophosphamide, Skin grafting

## Abstract

Leg ulcers as a complication of rheumatoid arthritis are difficult to heal. We present a case responding to treatment with cyclophosphamide and split skin grafting

## INTRODUCTION

Vasculitis involving small and medium-sized vessels is reported to be associated with rheumatoid arthritis. Arteritic leg ulcers are usually deep, punched out and slow healing.[1] We report a patient of rheumatoid arthritis with large necrotic ulcers who responded to cyclophosphamide pulse therapy and slough excision followed by split skin grafting.

## CASE REPORT

**Figure 1:**
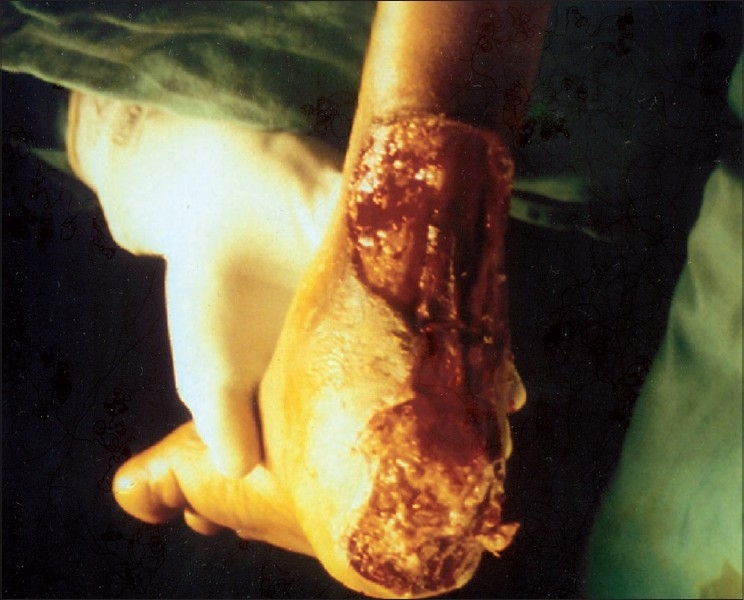
Large ulcer with slough over right heel and posterior aspect of ankle

A 38-year-old female with rheumatoid arthritis presented with ulcers of both legs of 1-month duration. She had gangrene of the left second, third and fourth toes. The ulcers were well defined with necrotic slough and haemorrhagic crusting involving right heel, posterior aspect of right ankle and calf of left leg [Figure 1]. The size of the ulcers varied from 2 to 6 cm. She was a diabetic on treatment with oral antidiabetics.

Investigations revealed hypochromic microcytic anaemia with neutrophilic leucocytosis and thrombocytosis. Rheumatoid factor was positive. ANA, DsDNA, antiSSA, antiSSB, antiScl 70, anticardiolipin antibody, antiJo-l, antiu1RNP and anticentromere A were negative. Biopsy of the ulcer showed evidence of leucocytoclastic vasculitis.

She received a single pulse of 500 mg of intravenous cyclophosphamide and antibiotic. She was then treated with 40 mg prednisolone, 50 mg of cyclophosphamide and oral antidiabetics.

**Figure 2:**
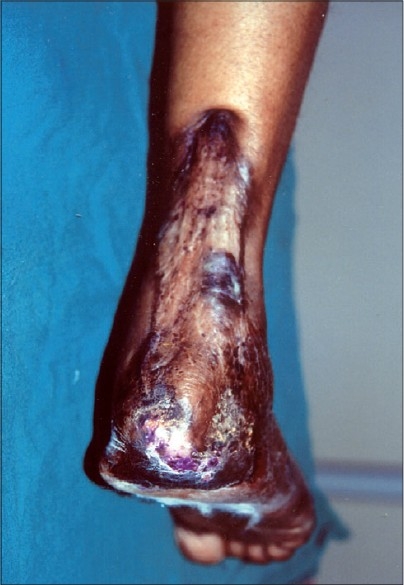
Complete healing of the ulcer after split skin grafting

A week later under general anaesthesia, necrotic slough was excised, gangrenous toes were amputated and split skin grafting placed over the ulcers. Pulse therapy with cyclophosphamide was repeated after 15 days since she developed two new ulcers. Approximately 50% of the graft had taken. Two weeks later split skin grafts were again placed over the ulcers and patient continued cyclophosphamide 50 mg while her steroids were gradually tapered and stopped. Ulcer healed over a period of 3 months [Figure 2]. She had no recurrence of ulcers in 10 months after surgery.

## DISCUSSION

Vasculitis associated with rheumatoid arthritis involves both small and medium-sized vessels and is associated with neuropathy, digital gangrene, nail fold infarct and palpable purpura. Skin may show necrotic painful ulcers with black scabs.[2] Gangrene results from vasculitic changes involving the digital vessels and involves hands and foot. Cyclophosphamide is used for potentially life-threatening dermatoses that are resistant to other forms of treatment. These include systemic necrotising vasculitis, severe forms of systemic lupus, severe blistering disorders, multi-centric reticulohistiocytosis, relapsing polychondritis and pyoderma gangrenosum.[3] Necrotic slough not only acts a nidus for infection but also impairs collagen synthesis as fibroblasts have to compete with bacteria and inflammatory cells for oxygen and nutrients. Breakdown of collagen is enhanced due to collagenolytic activity of mediators released to combat secondary infection.[4] Split skin grafting is useful for leg ulcers. Healing by secondary intention leads to contraction, deformities and scars. Split skin grafts protect the vital structures from injury, acts as a biological dressing and are a source of growth factor and interleukins.[5] The graft can be thin (0.006“-0.012”) intermediate (0.012“-0.018”) or thick (0.018“-0.024”). We opted for intermediate split skin graft as capillaries are more in comparison to other types of split skin grafts. Necrotic slough was removed to create a healthy ulcer bed for better uptake of intermediate split skin grafting. These steps helped us in achieving a functionally and cosmetically acceptable result.
